# One-Step Preparation of Phenyl Boron-Modified Magnetic Mesoporous Silica for Selective Enrichment of *cis*-Diol-Containing Substances

**DOI:** 10.3390/molecules23030603

**Published:** 2018-03-07

**Authors:** Hua Fu, Jing Hu, Min Zhang, Yuerong Wang, Hongyang Zhang, Ping Hu

**Affiliations:** 1 Shanghai Key Laboratory of Functional Materials Chemistry, School of Chemistry & Molecular Engineering, East China University of Science and Technology, Shanghai 200237, China; fuhua8711@126.com (H.F.); hjhuali2012@163.com (J.H.); wangyuerong@ecust.edu.cn (Y.W.); hongyang_zhang@ecust.edu.cn (H.Z.); 2 School of Chemical Engineering, Qinghai University, Xining 810016, China; 3 Shanghai Key Laboratory of New Drug Design, School of Pharmacy, East China University of Science and Technology, Shanghai 200237, China

**Keywords:** magnetic solid-phase extraction, one-step preparation, phenylboronic acid, *cis*-diol-containing substance, selective enrichment

## Abstract

For enrichment and separation of *cis*-diol-containing compounds from biomatrix, a new type of magnetic nanoparticles named MS-48-PBSC, whichwas facilely prepared in a one-step heterogeneous reaction. The morphology results demonstrated that the MS-48-PBSC was a spherical nanomaterial containing a core of silica-coated magnetic particle with a diameter of about 200 nm, and a cover layer of mesoporous silica with a thickness of approximate 50 nm. The characterization results showed that MS-48-PBSC presented a pore size of 4.2 nm, a surface area of 548 m^2^·g^−1^, and a pore volume of 0.30 cm^3^·g^−1^. The MS-48-PBSC also exhibited magnetism of 42 emu·g^−1^ that contributed to the easy separation of magnetic nanomaterial within 30 s from the matrix with the aid of the external magnetic field. In addition, the MS-48-PBSC exhibited high adsorption capacity for adenosine, xanthosine, uridine, sialic acid, and teicoplanin with 0.60, 0.51, 0.42, 0.75, and 1.26 mg/g, respectively, and showed a high selectivity for the *cis*-diol structure compounds, relative to interferences of bovine serum albumin, guanine, uric acid, and xanthine. The recoveries of adenosine, xanthosine, uridine, sialic acid, and teicoplanin were 71.8–114.1% with relative standard deviation (RSD) ≤ 8.6%, and the enrichment factors of them were 8–11. MS-48-PBSC exhibited quick separation capability from matrix, high adsorption capacity and size exclusion for bovine serum albumin, which could meet the requirements of separation and enrichment for substances with a *cis*-diol structure.

## 1. Introduction

*Cis*-diol-containing biomolecules, such as sugars, nucleosides, and catecholamines, are closely related to physiological functions and diseases and participate in various biochemical reactions and life activities [1,2]. The identification of these molecules is of considerable significance. Molecules of medicine, including aminoglycoside antibiotics, flavonoid glycosides, and saponins from natural sources, also contain the *cis*-diol function group [3]. The biomolecules and drug metabolites are widely distributed in tissue, blood, and urine [4,5,6]. Some of these substances are present at trace levels in complex matrices and difficult to detect directly. Thus, pretreatment technologies for enriching target compounds with *cis*-diol structures and eliminating matrix interference should be developed. Boron-group-functionalized materials have become the main solid-phase extractants in the separation and enrichment of *cis*-diol compounds [7]. Given the boronic acid group is at equilibrium between the undissociated and dissociated forms by a changing pH, the group can be used as selective adsorbent for *cis*-diol compounds. Under alkaline conditions, the boron atom is complexed with oxygen and changes from the original planar *sp*^2^ hybrid to the tetrahedral *sp*^3^ hybrid. The *cis*-boric acid ions simultaneously form a five- or six-membered cyclic ester with 1,2-*cis*-diols. When the condition becomes acidic, *sp*^3^ hybridizes back to the *sp*^2^ hybrid, and the cyclic ester dissociates [8,9,10]. Therefore, the separation and enrichment of *cis*-diol compounds from complex samples by using boron-affinity materials are highly selective and easy to operate.

To date, a variety of boron-affinity materials, such as boron-based polymer resins, molecularly imprinted materials, magnetic nanoparticles, silica particles, monolith columns, and graphenes have been developed. The materials have been widely applied in sample pretreatment, such as solid-phase extraction (SPE) and dispersive extraction–adsorption. Compared with other materials, the prominent feature of boron-based magnetic nanomaterials is that the solid–liquid separation can be simplified by applying a magnetic field. In addition, a higher extraction efficiency and faster desorption time can be achieved on nanoparticles compared with micron-sized particles due to the large surface area [11,12,13]. Xiong and coworkers [14] developed one type of boron-affinity magnetic nanoparticles. Ferroferric oxide magnetic nanoparticles, ethyl orthosilicate hydrolysates, and bifunctional-molecule-modified epoxy groups were used as core, cover layer, and surface functional groups, respectively. Amino phenylboronic acid was finally immobilized on the surface of the magnetic nanoparticles through a ring-opening reaction. This method utilized the easy modification of silica gel groups and thus became one of the most commonly used methods for the preparation of boron-affinity magnetic materials. The solvothermal method is another commonly used method for the preparation of magnetic nanoparticles by direct modification of specific active groups. Materials with specific active groups were subsequently directly grafted using boronic acid groups for the determination of biomolecules. Deng et al. [15] prepared amino-group-modified magnetic iron oxide nanoparticles by using the solvothermal method and then obtained the magnetic boron-affinity material by using a sequential two-step amide reaction with oxalyl chloride and amino phenylboronic acid. This magnetic boron-affinity material was suitable for glycoprotein separation. In recent years, in order to improve the extraction capacity of boron-based nanomaterials, researchers have increased the specific surface area of nanoparticles and the surface grafting rate of the functional group.

Magnetic mesoporous nanosilica (MMNS) materials contain a core of silica-coated magnetic practical and a cover layer of mesoporous nanosilica. As functional nanomaterials used for SPE, MMNS possesses an easily modifiable Si–OH on the surface and uniform mesopore structure for carrying targets. Pore size of MMNS is also easily adjustable for various applications. We propose that modification of the mesoporous silica surface by using phenylboron to increase the loading capacity of boron-affinity materials. In addition, the exterior mesoporous silica shell can also keep the macromolecular interference (such as bovine serum albumin (BSA)) in the matrices out of the interior pore walls through the adjustable mesopore diameters. This method improves the selectivity of boron-affinity materials to the small-molecule *cis*-*o*-dihydroxy compounds.

The most common MMNS were prepared using MCM-41 and SBA-15 as their exterior shells. The former material contains an exterior mesoporous shell within a confined molecular pore size of 2.3–3.8 nm. Such small pore sizes were unfavorable for immobilizing phenylboronic acid groups onto the surface of the magnetic mesoporous layer. The SBA-15 contains a mesoporous pore size within 8–16 nm. The pore size allowed phenylboronic acid groups to be immobilizated, but such large dimensions result in the absence of size exclusion capability for certain macromolecular compounds, such as chorionic gonadotropin (36.7 kDa, 7.5 nm × 3.5 nm × 3 nm), luteinizing hormone (approximately 30 kDa), and a thyroid stimulating hormone (approximately 25–28 kDa). This drawback lead to inevitable interference on the selective adsorption for small-molecule *cis*-diol compounds.

For the modification of phenylboron functions on the silicon surface, the common method was to heterogeneously modify the phenylboron to the silicon material through multi-step heterogeneous reaction. However, the yield of phenylboron-based modification is low. Obviously, various factors including reaction kinetics would affect heterogeneous reactions. In order to meet the challenge, Xu et al. [8] repeated the grafting reaction twice to improve the yield of the boronic acid functionalized FDU-12 mesoporous material. Xu and coworkers [16] modified the polyethyleneimine on the silicon surface to increase the amount of modification of the boron group with a sequential four-step heterogeneous amidation reaction. However, the purification process of the product consumed a lot of solvent and time to clean and centrifuge the nanometer-sized product, which would easily lead to the loss of materials.

In our previous work, a facile method was developed to prepare magnetic mesoporous silica nanoparticles with an average pore size of ~5.0 nm by using polyethylene glycol–polylactic acid copolymer as a template. Size exclusion for macromolecular compounds with a molecular weight of more than 20 kDa can be performed on this nanomaterial [17]. If these magnetic mesoporous nanoparticles are used to immobilize a phenylboronic acid silane coupling (PBSC) agent onto the surface of a magnetic mesoporous layer, then the obtained new magnetic nanoparticles will have appropriate pore size for size exclusion, large surface area for loading capacity, and magnetic performance for simple process of separation of high selectivity and high capacity adsorption for small-molecule *cis*-diol compounds in a complex matrix.

To prepare boron-affinity materials, the phenylboron group is normally immobilized on the silicon material surface step-by-step by heterogeneous reaction. The low yield limited the application of this method. In this work, we aimed to develop a simple and convenient approach to preparation of boronic-modified MMNS named MS-48-PBSC based on a coupled reaction of a prepared PBSC agent with Si–OH on the surface of MMSN. Then, we applied MS-48-PBSC to the adsorption and separation of small-molecule-containing *cis*-diol group. 

## 2. Results and Discussion

### 2.1. Synthesis of MS-48-PBSCP

**Scheme 1:**
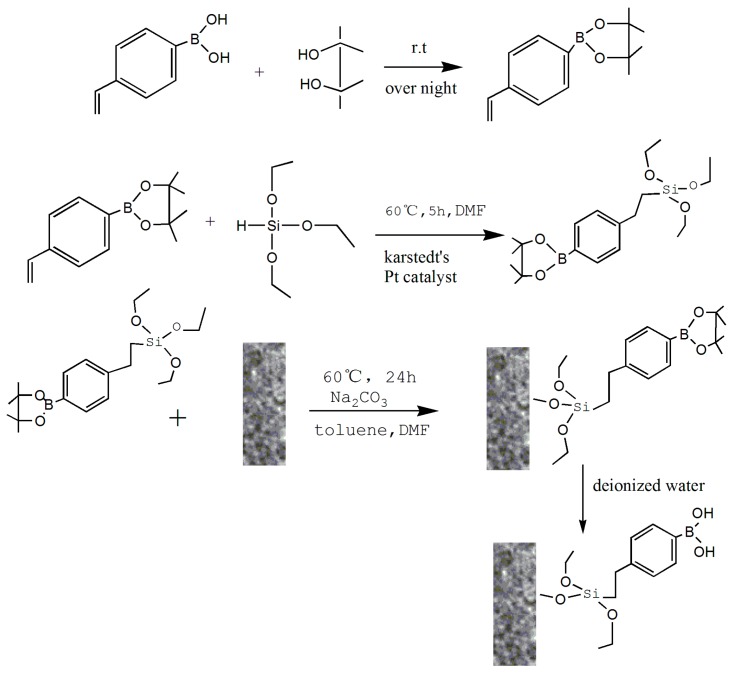

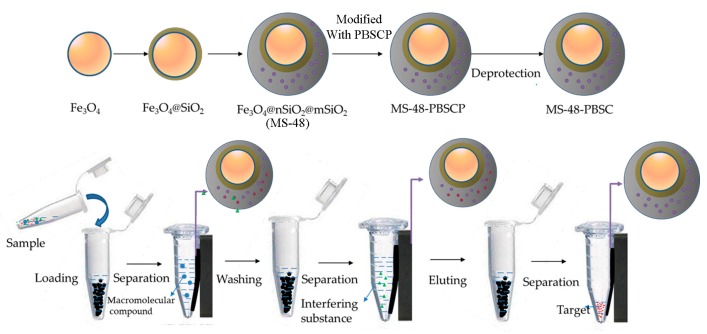
Scheme of the preparation of mesoporous boronic acid-functionalized MS-48-PBSC and its application in magnetic solid-phase extraction. 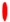 Target; 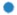 macromolecular compound; 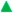 interfering substance; PBA, phenylboronic acid; MS-48-PBSCP, phenylboron modified magnetic mesoporous silica nanoparticles extractant.

The preparation and application of MS-48-PBSC is illustrated in Scheme 1.

Scheme 1 demonstrates the approach to preparation of MS-48-PBSC and its application process. First, we synthesized a silane-coupling agent PBSCP by hydrosilylation reaction with triethoxysilane and pinacol-protected 4-vinylbenzeneboronic acid as a starting reagent. The synthesis steps of PBSCP are shown at the top of the Scheme 1. Then, we applied the Fe_3_O_4_ particles as magnetic core and mesoporous material MS-48 as the outer cover, which has appropriate pore size with 4.7 nm to conjugate benzene boron silane coupling agent PBSCP. Finally, we used this new magnetic mesoporous material to extract and enrich several *cis*-diol-containing substances. The separation process is shown in the bottom of Scheme 1.

To prevent the introduced silane from affecting the stability of boron hydroxyl, we used a predesigned pinacol-protected 4-vinyl phenylboronic acid as an initial reactant to synthesize a benzene boron silane coupling agent with triethoxysilane through hydrosilylation reaction. Finally, MS-48-PBSCP was prepared using a coupled reaction that immobilized the PBSCP agent onto the surface of a magnetic mesoporous layer. 

The product of PBSCP contained two substitution isomers, which can react with hydroxyl on MS-48. Hence, it is not necessary to separate them after the reaction. PBSCP was then grafted using a coupling reaction between triethoxysilane and Si–OH in one step. The first advantage of the coupling reaction is the extremely mild conditions and facile operation. The second advantage is that the one-step method significantly improved the synthetic efficiency for the heterogeneous reaction, In addition, the approach can reduce the posttreatment steps, which can not only make the product treatment simple but also reduce the usage of organic regents.

**Figure 1:**
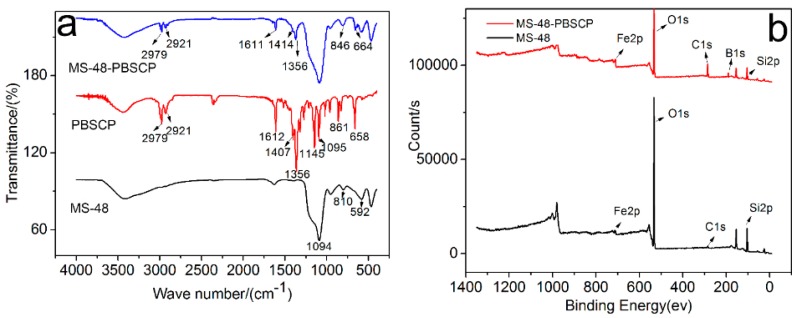
(**a**) Infrared spectroscopy of MS-48 (bottom), PBSCP (middle), and MS-48-PBSCP (top); (**b**) X-ray photoelectron spectroscopy curves of the mesoporous silica MS-48 and MS-48-PBSCP.

The infrared (IR) spectra (Figure 1a) of PBSCP and MS-48-PBSCP show bands centered at 2979, 2921, and 1356 cm^−1^, which are ascribed to the vibrations of adsorbed –CH_3_ and –CH_2_. The vibration at 3429 cm^−1^ is ascribed to –OH, and the vibration at 1611 cm^−1^ is ascribed to C=C from the benzene rings. These results imply that the PBSCP-bonded molecules are successfully grafted onto the MS-48 materials [18].

The X-ray photoelectron spectroscopy (XPS) spectra (Figure 1b) of MS-48-PBSCP (top) and MS-48 (bottom) showed peaks centered at 714, 533, 285, 191, and 104 eV, which are the characteristic peaks of Fe, O, C, B, and Si, respectively. The peak heights represent their relative content at the material surface. Evidently, MS-48-PBSCP displays a higher C peak than MS-48; the peak was derived from unhydrolyzed TEOS. The C and B content ratio in MS-48-PBSCP was in agreement with that of PBSCP, thus demonstrating the successful combination of PBSCP with the MS-48 surface.

### 2.2. Morphology of MS-48-PBSCP

**Figure 2:**
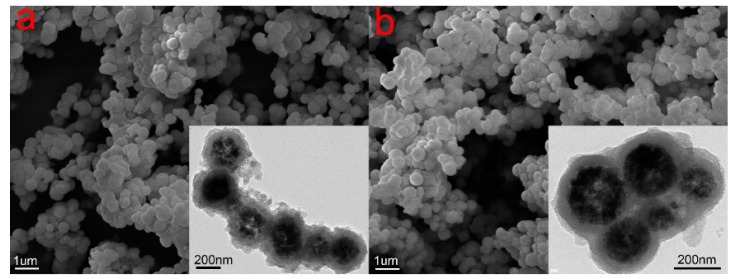
Scanning electron microscopy and Transmission electron microscopy (inset) images of (**a**) MS-48 and (**b**) MS-48-PBSCP.

The surface profiles of MS-48 and MS-48-PBSCP are illustrated in Figure 2a,b. According to the scanning electron microscopy (SEM) images, the obtained microspheres exhibited a narrow size distribution with a mean diameter of 250 nm, a spherical particle profile, uniform dispersion, and identical bubble size.

**Figure 3:**
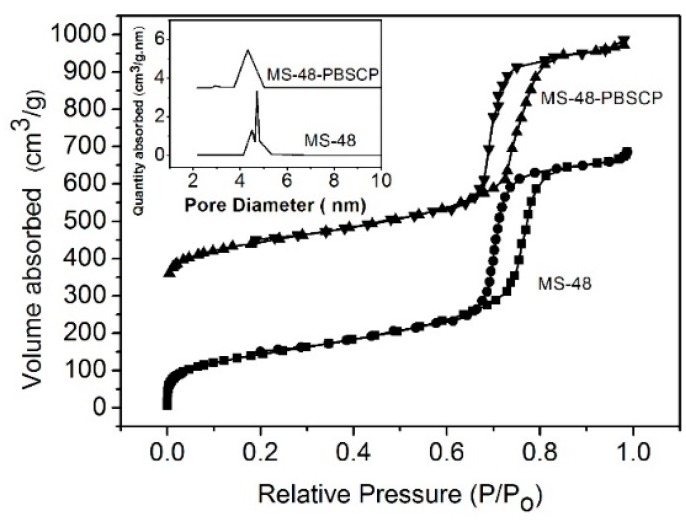
N_2_ sorption isotherm curves and pore size distribution plots for MS-48 and MS-48-PBSCP.

The transmission electron microscopy (TEM) images of MS-48 and MS-48-PBSCP (Figure 2a,b, respectively) show a magnetic core with a diameter of approximate 200 nm and porous silica shells with a thickness of approximate 50 nm. The TEM images further show that the mesostructure is retained after functionalization. The pore sizes are approximate 4.7 and 4.2 nm for MS-48 and MS-48-PBSCP, respectively, as derived from the adsorption branch by using the Barrett–Joyner–Halenda (BJH) method. MS-48-PBSCP exhibited a narrower pore size distribution than MS-48 by approximate 0.5 nm (Figure 3). This result implied that the size exclusion was effective for molecules with a size of approximate 4 nm, such as the hormone glycoproteins mentioned in the introduction. The Brunauer–Emmett–Teller (BET) surface area and total pore volume were 705 cm^2^∙g^−1^ and 0.38 cm^3^∙g^−1^ for MS-48 and 548 cm^2^∙g^−1^ and 0.30 cm^3^∙g^−1^ for MS-48-PBSCP, respectively. This result implied that even after functionalization, the MS-48-PBSCP material retained its high porosity compared with MS-48. Moreover, the result suggested that the dense pore channels in the shell were exits to the microsphere surface, which improved the extraction capacity of the material. 

**Figure 4:**
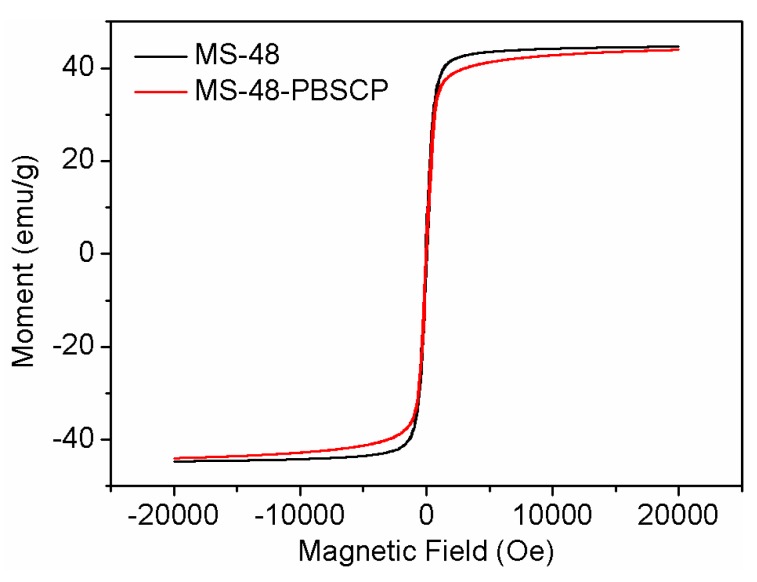
Room-temperature magnetic hysteresis loops of MS-48 and MS-48-PBSCP.

The saturation magnetization values of MS-48 and MS-48-PBSCP were approximate 44 and 42 emu∙g^−1^ (Figure 4). When suspended homogeneously in water, both MS-48 and MS-48-PBSCP exhibited fast sedimentation under the applied magnetic field in 30 s. Hence, it was indicated that MS-48-PBSC had the fast separation performance.

### 2.3. pH Investigation of Adsorption and Elution

**Figure 5:**
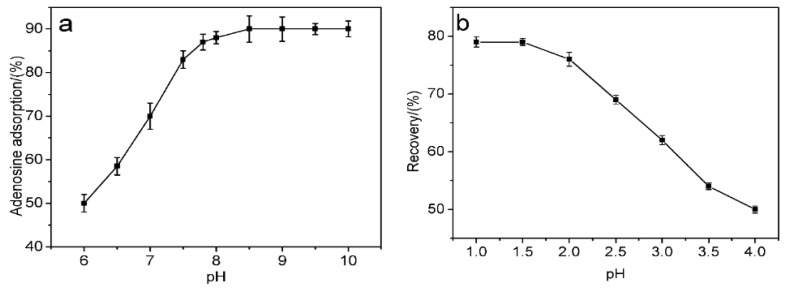
Effect of pH on MS-48-PBSC adsorption (*n* = 3). (**a**) Loading with adenosine in alkalinity buffer and (**b**) Eluting adenosine in acidic solution (*n* = 3).

Binding pH is an essential factor for boronate to selectively adsorb or elute *cis*-diol structures. Boronate and *cis*-diol can complex to form a lactone structure under alkaline conditions and release *cis*-diols under acidic conditions. Notably, a low pH is a critical factor for the boronate affinity of *cis*-diol-containing biomolecules to ensure biological stability. The absorption capacities of MS-48-PBSC for adenosine at different pH values were therefore measured (Figure 5). Phosphate buffer saline (PBS) was used to adjust the pH in the 6.0–8.5 range, and hydrochloric acid–Tris buffer was used to adjust the pH in the 8.5 to 10.0 range. Upon the addition of adenosine (Figure 5a), the adsorption capacity during supernatant adsorption increased with increasing pH. However, when the pH exceeded 7.8, no evident change was observed. We therefore selected pH = 7.8 as the best binding pH. The alkalescence adsorption condition indicated that MS-48-PBSC was fit for binding *cis*-diol-containing biomolecules, which usually exist in a neutral environment. During desorption (Figure 5b), we used formic acid from pH 1.0 to 4.0 to elute the samples. Formic acid at pH 1.5 produced optimum results. Therefore, we selected a formic acid solution with pH = 1.5 plus 40% methanol as the optimum elution conditions.

### 2.4. Adsorption Capacity Investigation

**Figure 6:**
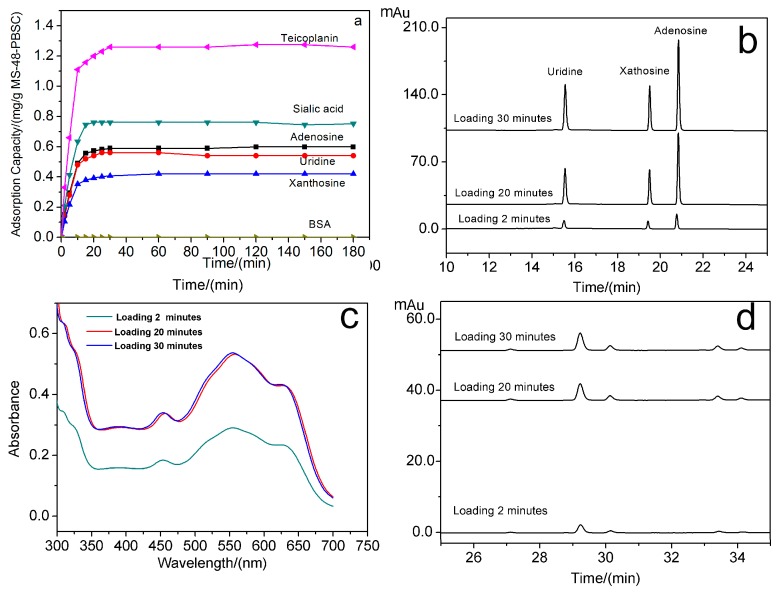
(**a**) Adsorption capacity at different loading times for uridine, xanthosine, adenosine, sialic acid, teicoplanin, and bovine serum albumin; (**b**) High-performance liquid chromatograph of the elution of xanthosine, uridine, and adenosine with different loading times; (**c**) Ultraviolet absorption spectrum of eluted sialic acid with different loading times; (**d**) High-performance liquid chromatograph of the elution of teicoplanin with different loading times.

We selected uridine, xanthosine, adenosine, sialic acid, teicoplanin as model compounds for nucleosides, monosaccharides and glycopeptide antibiotics in *cis*-diol-containing biomolecules to test the extraction performance of MS-48-PBSC. The adsorption capacity for targets was determined using high-performance liquid chromatograph (HPLC) and ultraviolet (UV)-visible (vis) spectrophotometry. All testing results are shown in Figure 6a–d. The loading time for the binding of uridine, xanthosine, adenosine, sialic acid, teicoplanin, and BSA to MS-48-PBSC was investigated. The results are shown in Figure 6a. Figure 6b shows the HPLC of elution of uridine, xanthosine, and adenosine with loading times of 2, 20, and 30 min. Figure 6c shows the UV absorption spectrum of eluted sialic acid with loading times of 2, 20, and 30 min, and Figure 6d is the HPLC of eluted teicoplanin with loading times of 2, 20, and 30 min. Following the addition of the tested samples to MS-48-PBSC, (Figure 6a–d), the absorption of all samples except for BSA rapidly increased and reached a maximum value within 30 min. This result implied the rapid and stable bindings between *cis*-diols and MS-48-PBSC. Such characteristics are important features for high-throughput detection of *cis*-diol structures. The maximum adsorption amount of MS-48-PBSC reached 0.60, 0.54, 0.42, 0.75, and 1.26 mg·g^−1^ for adenosine, xanthosine, uridine, sialic acid, and teicoplanin, respectively. 

We applied a commercial magnetic nanoparticle prepared by coating a boron-based polymer onto the outer layer of the magnetic core for comparison. The maximum adsorption amount of commercial nanoparticle reached 0.36, 0.48, 0.51, 0.54, and 1.02 mg·g^−1^ for adenosine, xanthosine, uridine, sialic acid, and teicoplanin, respectively. These results showed that the adsorption capacity of MS-48-PBSC was similar to that of the commercial material.

### 2.5. Selectivity Investigation

We also selected BSA as biological macromolecule interference and guanine, uric acid, and xanthine as small biological molecule interferences. The selectivity test results showed the MS-48-PBSC exhibited no adsorption toward BSA (Figure 6a) because BSA cannot enter the channel of MS-48-PBSC owing to the size exclusion effect. This outcome implied that the small pore entrance size of approximate 4.2 nm enabled the exclusion of macromolecule proteins. 

**Figure 7:**
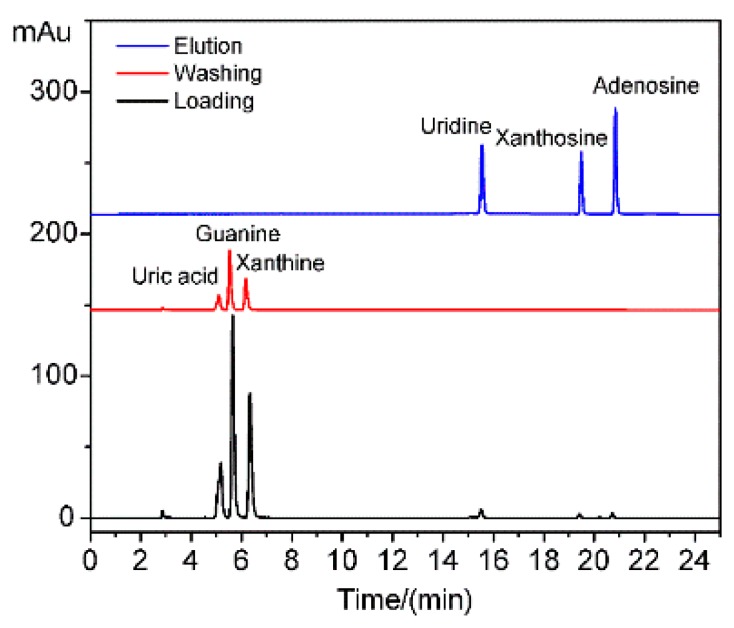
Selectivity toward uridine, xanthosine, adenosine, guanine, uric acid, and xanthine.

The HPLC of the supernatants of uridine, xanthosine, adenosine, guanine, uric acid, and xanthine during loading, washing, and elution are shown in Figure 7. The small biological molecule interferences of guanine, uric acid, and xanthine without *cis*-diol structures existed in the loading and washing supernatants but were absent in the elution solution. Thus, the interferences were weakly adsorbed by the porous structure and easily desorbed during the washing step. By contrast, the targets of uridine, xanthosine, and adenosine with *cis*-diol structures were detected in the elution solution but not in the loading and washing supernatants. Thus, the materials exhibited high selectivity toward *cis*-diol structures due to the complex reaction. High selectivity was also derived from the mesoporous structure exclusion. This feature is beneficial for the application of MS-48-PBSC in complex biological samples, such as blood, urine, and tissue.

### 2.6. Recovery and Enrichment Times

The recovery of the test objects in Section 2.4 enriched by the MS-48-PBSC was investigated and the results are listed in Table 1. The recovery of three concentration levels of uridine, xanthosine, adenosine, sialic acid and teicoprazole were 71.8–114.1% with RSD 1.5–8.6%. The results suggested that the MS-48-PBSC could be used for separation and enrichment of *cis*-diol-containing compounds. 

The concentrations of the five model compounds were increased after enrichment by MS-48-PBSC. The enrichment factor reached 10, 11, 9, 10, and 9 corresponding to uridine, xanthosine, adenosine, sialic acid and teicoplanin respectively. After enrichment, the detection limits of *cis*-diol-contaning substances were decreased.

### 2.7. Comparative Results for the Different Methods

The preparation method for MS-48-PBSC proposed in this paper has advantages in minimization of processing time and solvent consumption compared to those of heterogeneous reaction products containing at least two steps. This was attributed to the one-step preparation approach of MS-48-PBSC reduce the posttreatment steps. In extraction and enrichment processes, the MSPE method only used an external magnetic field for separation and enrichment. On one hand, it reduced the costs of the solid-phase extraction cartridge. On the other hand, the solvent usage of 1 mL for MS-48-PBSC method was less than that of the SPE method with at least 3–5 mL. Therefore, the MS-48-PBSC was more promising than the nanomaterials prepared with numerous heterogeneous reaction steps for extraction and enrichment of *cis*-diol-containing compounds.

## 3. Materials and Methods

FeCl_3_ (99.9%), ethylene glycol (98%), and sodium acetate (99.9%) were obtained from Sinopharm Chemical Reagent Co. Ltd., Shanghai, China. Pluronic polyethylene glycol–polylactic acid (PEG4000-PLA800 (P848)) was obtained from Jinan Daigang Co. Ltd., Jinan, China; tetraethoxysilane (TEOS, 98%), triethoxysilane (97%), pinacol (99%) and 4-vinyl phenylboronicacid (98%) bovine serum albumin (98%), sialic acid (98%), teicoplanin (98%), uridine, xanthosine, adenosine, guanine, uric acid, and xanthine standards were obtained from Energy Chemica, Shanghai, China; pyrocatechol (99.9%), hydroquinone (99.9%), and trifluoroacetic acid were obtained from Shanghai Ling Feng Chemical Reagent Co. Ltd., Shanghai, China; Karstedt’s catalyst was obtained from Alfa Aesar, Shanghai, China, commercial phenylboron modified magnetic nanomaterials were obtained from Enriching Biotechnology Ltd., Shanghai, China. All chemicals and solvents were used without further purification. N_2_ adsorption-desorption isotherms were measured at 196 °C using ASAP 2010 gas adsorption apparatus (Micromeritics Instrument Co., Norcross, GA, USA). Field emission scanning electron microscopy (FE-SEM) was performed on an FEI Magellan 400 electron microscope (Hillsboro, OR, USA). Transmission electron microscopy (TEM) images were taken with a JEOL JEM-2100 microscope operated at 200 kV (Tokyo, Japan). The magnetic properties were determined with a physical property measurement system (PMS-9T (EC-II), Quantum Design Company of USA, San Diego, CA, USA). Fourier-transform infrared (FT-IR) spectrometry was carried out using a Nicolet 380 FT-IR spectrometer (Thermo Fisher Scientific, Waltham, MA, USA). Nuclear magnetic resonance (NMR) spectra was measured using an Agilent Technologies 400 MR (Santa Clara, CA, USA). Exact mass was measured with a Waters ACQUITY TQD Electrospray ionization mass spectrometry (ESI-MS) (Milford, MA, USA). Chromatography detection was carried out using a Shimadzu LC-20AT high-performance liquid chromatograph (Tokyo, Japan).

### 3.1. Synthesis of Mesoporous Silica-Coated Magnetic Oxide (MS-48)

Highly water-dispersible Fe_3_O_4_ nanospheres were prepared using the solvothermal reaction reported by Zhao et al. [19] Fe_3_O_4_@SiO_2_ microspheres were prepared using sol–gel method [20]. The core–shell-structured MS-48 composites were prepared using the nonionic diblock copolymer Pluronic^®^ PEG(4000)–PLA(800) (P48), developed by our laboratory [16] as template. MS-48 particles with ~4.7 nm pore diameters were obtained after being refluxed in ethanol solution at 60 °C for 48 h to remove the templates, and the reflux was repeated for three times. 

### 3.2. Synthesis of Mesoporous Boronic-Acid-Functionalized Magnetic Oxide (MS-48-PBSCP)

4-Vinyl benzene boronic acid pinacol ester was prepared according to the literature [21,22]. Specifically, a mixture of 4-vinyl phenylboronic acid (0.5 g) and pinacol (0.44 g) in 100 mL of dichloromethane in the presence of 20 g of 4A molecular sieves was stirred at 40 °C for 3 h to give a near quantitative yield of 4-vinyl benzene boronic acid pinacol ester. Analytical data for ^1^H-NMR of 4-vinyl benzene boronic acid pinacol ester is given as ^1^H-NMR (CDCl_3_): δ: 1.36 (s, 12H), 5.31 (d, 1H, *J* = 8 Hz), 5.83 (d, 1H, *J* = 16.0 Hz), 6.7 (dd, 1H), 7.41 (d, 2H, *J* = 8 Hz), 7.77 (d, 2H, *J* = 8 Hz); ^13^C-NMR (CDCl_3_): δ: 140.17, 136.84, 134.98, 125.46, 114.79, 114.77, 83.71, 77.00, 24.82; ESI-MS (M + H)^+^: *m*/*z* = 231.1. Analytical data for ^1^H-NMR of the PBSCP is given as ^1^H-NMR (CDCl_3_): δ: 0.91 (m, 2H), 1.17 (dt, 9H, *J* = 14 Hz and *J* = 6 Hz), 1.38 (s, 12H), 2.71 (m, 2H), 3.68 (dm, 6H), 7.22 (m, 2H, *J* = 8 Hz), 7.68 (d, 2H, *J* = 12 Hz); ^13^C-NMR (CDCl_3_): δ: 134.93, 136.84, 129.02, 128.21, 127.35, 127.24, 83.58, 77.06, 58.82, 58.41, 29.13, 24.85, 18.33, 12.44. ESI-MS (M + Na)^+^: *m*/*z* = 417.2.

PBSCP was prepared according to the literature [23,24,25]. Specifically, 0.95 g of 4—vinyl benzene boric acid pinacol ester and 300 ppm of Karstedt’s catalyst were dissolved in 5 mL of drying *N*,*N*-dimethylformamide (DMF). After the mixture was activated for 20 min at 50 °C, 0.82 g of triethoxysilane was added dropwise to the solution and stirred for 5 h at 60 °C under the protection of nitrogen. Finally, the DMF and excess triethoxysilane were removed under vacuum, and the nearly equivalent amount of PBSCP as a colorless oil was obtained.

MS-48 (0.15 g) and 0.2 g of Na_2_CO_3_ were dispersed in 20 mL of a mixed solution of toluene and DMF (*v*:*v* = 2:3). After ultrasonic treatment for 30 min, 0.5 g of PBSCP was added to the solution. The mixture was heated to 60 °C for 24 h with magnetic stirring under the protection of nitrogen. The products were collected with a magnet and washed twice by DMF followed by three washings with toluene. Finally, product MS-48-PBSCP was dried at 60 °C overnight.

### 3.3. Removal of the Pinacol Protecting Group from the MS-48-PBSCP

Prior to extraction, MS-48-PBSCP was used to remove the pinacol-protecting group. In brief, 100 mg of MS-48-PBSCP was added into 3 mL of 1 mol L^−1^ formic acid and methanol mix solution (*v*:*v* = 1:1) for 30 min of sonication dispersion and vortex mixing. After isolation using a magnet, the supernatant with the pinacol protecting group was abandoned. Finally, the unprotected magnetic nanoparticle product MS-48-PBSC was washed with 3 mL of 50% methanol solution several times and 1 mL of phosphate-buffered saline (PBS) (pH = 7.8) several times.

### 3.4. pH Optimization of the Extraction and Desorption 

The pH optimization of the extraction and desorption were investigated according to the literature [16]. Typically, an adenosine solution at 20 μg·mL^−1^ was selected as the test sample. In the loading process, pH of the adenosine solution was adjusted with PBS buffer or hydrochloric acid–tris buffer. After interacted with MS-48-PBSC nanoparticles for 30 s with the aids of ultrasonic and vortex mixing, the magnetic nanoparticles were isolated by a magnet and the supernatant was detected via HPLC. During the elution, adenosine-adsorbed MS-48-PBSC was eluted with different concentrations of formic acid solution and 40% methanol as elution solvent. The desorption time varies from 2 min to 3 h, and the elutes were detected via HPLC.

### 3.5. Enrichment of cis-Diol-Containing Compounds

Adenosine, xanthosine, and uridine solutions were used as the typical nucleoside samples, sialic acid as the monosaccharide sample, teicoplanin as the antibiotic sample, and BSA as the macromolecular sample. Small biological molecules guanine, uric acid, and xanthine were selected as interferences samples. All samples were prepared as stock solutions at 1 mg·mL^−1^. 

For evaluation of the adsorption capacity of MS-48-PBSC, the above-mentioned cis-diol structure-containing samples except teicoplanin and BSA were formulated into 100 μg·mL^−1^ (The concentration of teicoplanin was 200 μg·mL^−1^) solutions with PBS (pH = 7.8) buffer, respectively, and then 100 mg MS-48-PBSC was added. The loading and elution processes were performed using the optimal conditions listed in Section 3.4. For the washing process, 3 mL of PBS (pH = 7.8) and acetonitrile solution (*v*:*v* = 90:10) were added, and ultrasonic and magnetic separation steps were repeated. Finally, the supernatants were diluted 3-fold and detected using HPLC and UV-Vis spectrophotometer. Benzene boron modified commodity nanomaterials was activated with PBS buffer (pH = 8.5) and 5% of cetyltrimethylammonium bromide in methanol/H_2_O (*v*/*v*, 1:1). The following operation was similar to the method used for MS-48-PBSC. 

For evaluation of the selectivity to *cis*-diol-containing compounds, the 1 mL solution included all of the above nucleoside samples, and small biological molecules interferences samples at 1.7 μg·mL^−1^ with pH = 7.8, and 60 mg MS-48-PBSC was added. The eluate is finally concentrated to 100 μL. The loading, washing, and eluting processes were performed using the above optimal conditions. Then, the concentrated eluates were detected using HPLC and UV-Vis spectrophotometer.

For evaluation of the recovery and enrichment times, we used three nucleoside standards, sialic acid and teicoplanin as the test samples. All of them were mixed in the three quality levels at 0.5, 5, and 20 μg respectively. Finally, the mixed *cis*-diol structure sample supernatants were detected using HPLC and UV-Vis spectrophotometer.

### 3.6. HPLC and UV-Vis Methods

The chromatographic column type was a VP-ODS C18 (5 µm, 4.6 mm × 200 mm, Shimadzu). The mobile phase was methanol: 0.2% acetic acid solution (*v*:*v* = 30:70), the wavelength of diode array detector for HPLC was 260 nm, the sample size was 10 µL, and the flow rate was 1 mL·mL^−1^.

BSA sample supernatants were diluted to 10:1 and detected using ultraviolet (UV)–visible (vis) spectrophotometry at 280 nm.

Sialic acid sample supernatants were detected using a procedure reported in the literature [26]. In brief, 100 μL of supernatant was added to 2 mL of a resorcinol–hydrochloric acid solution. After boiling for 30 min, the mixture was treated in an ice-bath for 3 min. Then, 4 mL of butyl acetate–butanol extract solution (*v*:*v* = 4:1) was added to extract the derivative. The derivative was detected using UV–vis spectrophotometry at 580 nm. The resorcinol–hydrochloric acid solution was prepared as follows: 20 mL of 8 mol·L^−1^ HCl solution, 62.5 μL of 0.1 mol·L^−1^ CuSO_4_ solution, and 2 mL of 181.6 mmol·L^−1^ resorcinol solution were mixed together and diluted with deionized water to 25 mL.

Teicoplanin sample supernatants were detected according to the literature [27] by HPLC. The chromatographic column type was VP-ODS C18 (5 µm, 4.6 mm × 200 mm, Shimadzu). Mobile phase A was PBS (pH = 6.0) and acetonitrile solution at 85:15 (*v*:*v*), while mobile phase B was PBS (pH = 6.0) and acetonitrile solution at 30:70 (*v*:*v*). The diode array detector wavelength selection of HPLC was 260 nm, the sample size was 10 µL, and the flow rate was 1 mL/min. The elution gradient conditions for the LC mobile phase were based on A/B from 100/0 (*v*/*v*) to 70/30 (*v*/*v*) at 32 min and 62.5/37.5 (*v*/*v*) at 32 min. The total chromatographic run duration was 35 min.

## 4. Conclusions

A new mesoporous material (MS-48-PBSC) for detecting *cis*-diol biological substances was developed. MS-48-PBSC with mesoporous silicon-coated Fe_3_O_4_@SiO_2_ as the nucleus resulted applying in a material with a high specific surface area. The mesoporous size exclusion effect enabled the exclusion of macromolecular proteins, which is beneficial for complex biological samples. Magnetism allows the easy separation of MS-48-PBSC from a complex matrix through an external magnetic field and reuse of material. During material preparation, the one-step immobilization of phenylboronic acid groups on the mesoporous wall allowed for an easy, time-efficient operation. Moreover, the phenylboronic acid group-modified mesoporous material can function at a relatively low pH range and improve the binding selectivity and affinity toward *cis*-diol structures. The method can be further applied to enrich of sugar complexes with *cis*-diol structures in biological samples.

## Figures and Tables

**Table 1:** Recovery of the uridine, xanthosine, adenosine, sialic acid, and teicoplanin.

Sample	The Added Amount (µg)	The found Amount (µg)	Recovery (%)	RSD (%, *n* = 3)
Uridine	0.533	0.407	76.4	4.4
	5.421	4.315	79.6	6.5
	20.09	16.19	80.6	5.7
Xanthosine	0.521	0.374	71.8	4.4
	5.222	3.765	72.1	1.5
	20.31	14.97	73.7	5.7
Adenosine	0.493	0.423	85.9	4.4
	5.279	4.413	83.6	6.5
	20.20	18.01	89.2	5.7
Sialic acid	0.516	0.524	101.6	8.4
	5.178	4.287	82.8	6.7
	20.36	19.73	96.9	7.7
Teicoplanin	0.547	0.482	88.2	7.9
	5.327	6.078	114.1	8.6
	20.16	19.13	94.9	6.2
